# ACTIONS TO REDUCE RISK OF OLDER ADULTS DURING EXTREME WEATHER EVENTS THAT THREATEN HEALTH AND LIFE

**DOI:** 10.1093/geroni/igz038.1990

**Published:** 2019-11-08

**Authors:** Lisa M Brown

**Affiliations:** Palo Alto University, Plo Alto, California, United States

## Abstract

Climate change places many older adults at unique risk for adverse, potentially life-threatening consequences. Compared to younger adults, older adults are more susceptible to the effects of extreme weather events and at greater risk for heat stroke and hypothermia. With advanced age, some people have vulnerabilities, such as social isolation and physical and mental health conditions that predispose them to a greater level of danger. Loss of power, that leaves people without functioning cooling or heating systems, can cause death because it exacerbates preexisting medical conditions and impairs thermoregulatory function for those with medical illnesses or taking certain medications. Many older adults who function independently may not self-identify as being vulnerable and take necessary precautions to remain safe. This presentation will describe recent research that found that many older adults are unaware of hypothermia and hyperthermia symptoms and the actions needed to maintain health and life.

